# Photons, photon jets, and dark photons at 750 GeV and beyond

**DOI:** 10.1140/epjc/s10052-016-4127-4

**Published:** 2016-05-18

**Authors:** Basudeb Dasgupta, Joachim Kopp, Pedro Schwaller

**Affiliations:** 1 Tata Institute of Fundamental Research, Homi Bhabha Road, Mumbai, 400005 India; 2 PRISMA Cluster of Excellence, Mainz Institute for Theoretical Physics, Johannes Gutenberg University, Staudingerweg 7, Mainz, 55099 Germany; 3 DESY, Notkestrasse 85, 22607 Hamburg, Germany

## Abstract

In new physics searches involving photons at the LHC, one challenge is to distinguish scenarios with isolated photons from models leading to “photon jets”. For instance, in the context of the 750 GeV diphoton excess, it was pointed out that a true diphoton resonance $S \rightarrow \gamma \gamma$ can be mimicked by a process of the form $p p \rightarrow S \rightarrow a a \rightarrow 4\gamma$, where *S* is a new scalar with a mass of 750 GeV and *a* is a light pseudoscalar decaying to two collinear photons. Photon jets can be distinguished from isolated photons by exploiting the fact that a large fraction of photons convert to an $e^+e^-$ pair inside the inner detector. In this note, we quantify this discrimination power, and we study how the sensitivity of future searches differs for photon jets compared to isolated photons. We also investigate how our results depend on the lifetime of the particle(s) decaying to the photon jet. Finally, we discuss the extension to $S\rightarrow A^\prime A^\prime \rightarrow e^+e^-e^+e^-$, where there are no photons at all but the dark photon $A^\prime$ decays to $e^+e^-$ pairs. Our results will be useful in future studies of the putative 750 GeV signal, but also more generally in any new physics search involving hard photons.

## Introduction

In their recent end-of-year jamboree, the ATLAS and CMS collaborations have reported an impressive cornucopia of LHC Run II results. One of them – a possible excess in the two photon final state at an invariant mass $\sim$750 GeV [1, 2] – has caused a flurry of discussion in the community [3]. Most of these works introduce a new neutral scalar particle $\phi$ with a mass around 750 GeV and decaying to two photons. Both the production and the decay of this particle typically proceed through loop diagrams. Constraints from Run I data imply that the production cross section of $\phi$ must be significantly larger at the Run II center-of-mass energy of 13 TeV than at the Run I energy of 8 TeV. Moreover, decay modes of $\phi$ other than $\phi \rightarrow \gamma \gamma$ should not be too strong, but at the same time, $\phi$ should have a large total width $\sim$45 GeV to optimally fit the data.

A very appealing class of alternative models explaining the 750 GeV excess are those in which the final state is in fact not two body, but contains two “photon jets”, i.e. groups of highly collinear photons [4–10]. If the photon jets are sufficiently collimated, they are indistinguishable from isolated photons using information from the electromagnetic calorimeter alone. Therefore, models of this type could explain the diphoton anomaly, as discussed in Refs. [11–16]. While the experiments have strong discriminating variables to reject e.g. photon pairs coming from neutral hadron decays, the studies [11–16] show that there are regions of parameter space where the photon jets are expected to pass the tight photon selection.

**Fig. 1:**
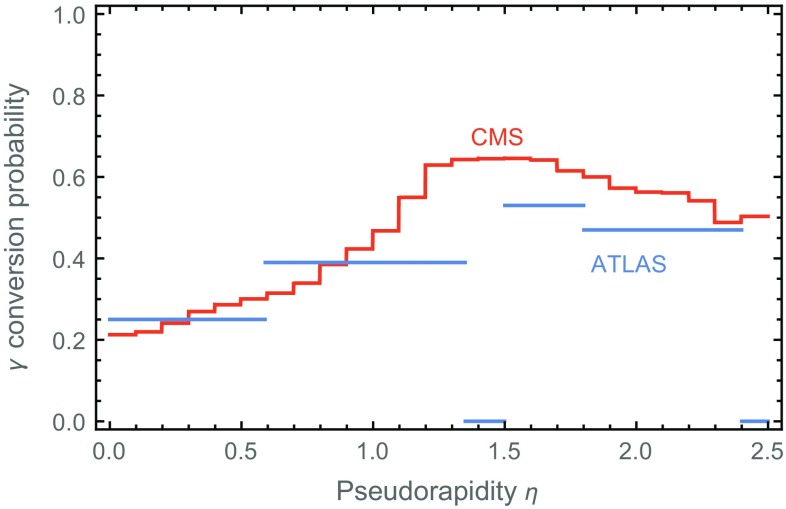
Probability for prompt photons to be reconstructed as converted photons in the ATLAS (*blue*) and CMS (*red*) detectors, based on 13 TeV data (ATLAS, Ref. [17]) and 8 TeV data (CMS, Ref. [18])

However, there is a catch: since photons have to travel through some amount of detector material before reaching the calorimeter, they have a high (e.g. $\sim$40 % at ATLAS [17]) probability of converting to an $e^+ e^-$ pair already in the inner detector, with nontrivial pseudorapidity dependence (see Fig. 1). Such conversions can occur in the strong electric field of an atomic nucleus through a process $\gamma + Z \rightarrow Z + e^+ + e^-$. “Converted photons” are routinely included in analyses involving photons.

For a high-$p_T$ photon jet with $\ge$2 photons, it is clear that the probability that at least one of the photons inside the jet converts is higher than for isolated photons. Even if no further discrimination is performed, we will show below that the ratio of converted to unconverted photon events already provides a powerful discrimination between isolated photon and photon jet models. Furthermore this ratio can also be used to improve the sensitivity of searches for photon jet events.

Going beyond conversion ratios, several other observables could be used to reveal the photon jet origin of signals involving photons, including non-resonant photons. This includes a mismatch of the track $p_T$ and the calorimeter $E_T$ if only one photon converts, a non-standard response of the signal to changes of the photon selection criteria, and converted photon candidates with more than two tracks.

In the rest of this note, we will first discuss the use of converted photon ratios to discriminate events with photon jets from isolated photons and to improve the sensitivity of searches for such models (Sects. 2 and 3). After that we will analyze the effects of finite lifetime of the intermediate states on this analysis (Sect. 4), and we will extend the discussion to models with dark photons decaying directly to displaced $e^+ e^-$ pairs (Sect. 5). Finally, we discuss the prospects of other observables in more detail (Sect. 6). While most of our numerical results are obtained using ATLAS 13 TeV data, we expect that at CMS similar results can be expected, since the conversion rate is similar in magnitude and rapidity dependence, as seen from Fig. 1.

## Photon jets

Before digging into the details, let us first review the type of models that can give rise to photon jets and which therefore can be probed by the methods we present below. Any particle that decays to two or more photons can produce a photon jet if it is sufficiently boosted. Consider a particle *a* with mass $m_a$ and Lorentz boost $\gamma = E /m_a$ decaying to two photons. The minimal opening angle between the two photons is

1 $$\begin{aligned} \Delta \phi _\mathrm{min}&= \arccos \left( 1- \frac{2}{\gamma ^2} \right) \approx \frac{2}{\gamma }. \end{aligned}$$

Experimentally, photon pairs with opening angles below $\Delta R \sim 0.01$ are difficult to distinguish from isolated photons in the calorimeter. Therefore if *a* is produced in the decay of a TeV scale resonance, one finds that for $m_a \lesssim 2$ GeV the photon pairs from each *a* decay can easily pass as isolated photon candidates.^1^ Models of this type were considered before in the context of exotic Higgs decays [4, 9, 10] and more recently as alternative interpretations of the 750 GeV resonance [11–16].

Couplings of a light state *a* to photons are also constrained by low energy data [19–25]. This makes it impossible to choose $m_a$ arbitrarily small. Nevertheless, $m_a$ could be so small that its (laboratory frame) decay length becomes comparable to or even larger than the size of the ATLAS and CMS inner detectors (about a meter). If *a* decays to $\gamma \gamma$ at a macroscopic distance from the beam pipe, but still within the inner detector, the two photons have a smaller conversion probability than for quasi-instantaneous *a* decay. We will consider this possibility in Sect. 4. If the decay length of *a* is so large that most decays occur outside the electromagnetic calorimeter, they can no longer mimic isolated photons. Such scenarios are, however, still of phenomenological interest in the context of displaced object searches, which look for objects decaying in the calorimeters or in the muon system [26, 27].

To be as model independent as possible, we will consider scenarios where a resonance *X* is produced in proton–proton collisions and decays to two light particles $a_1$, $a_2$, each of which in turn decays to $N_i$ photons:

2 $$\begin{aligned} p p \rightarrow X \rightarrow (a_1 \rightarrow N_1 \gamma ) + (a_2 \rightarrow N_2 \gamma ). \end{aligned}$$

As a concrete realization, consider the case of a scalar resonance *S* with loop induced couplings to gluons and tree level couplings to a light pseudoscalar *a*, which in turn couples to photons:

3 $$\begin{aligned} \mathcal{L} \supset \!-\!M_S^2 S^2 \!- \!m_a^2 a^2 \!+\! \frac{1}{\Lambda } S G_{\mu \nu } G^{\mu \nu } \!+ \!\lambda S a a + \frac{1}{f} a F_{\mu \nu }{\tilde{F}}^{\mu \nu }. \end{aligned}$$

**Fig. 2:**
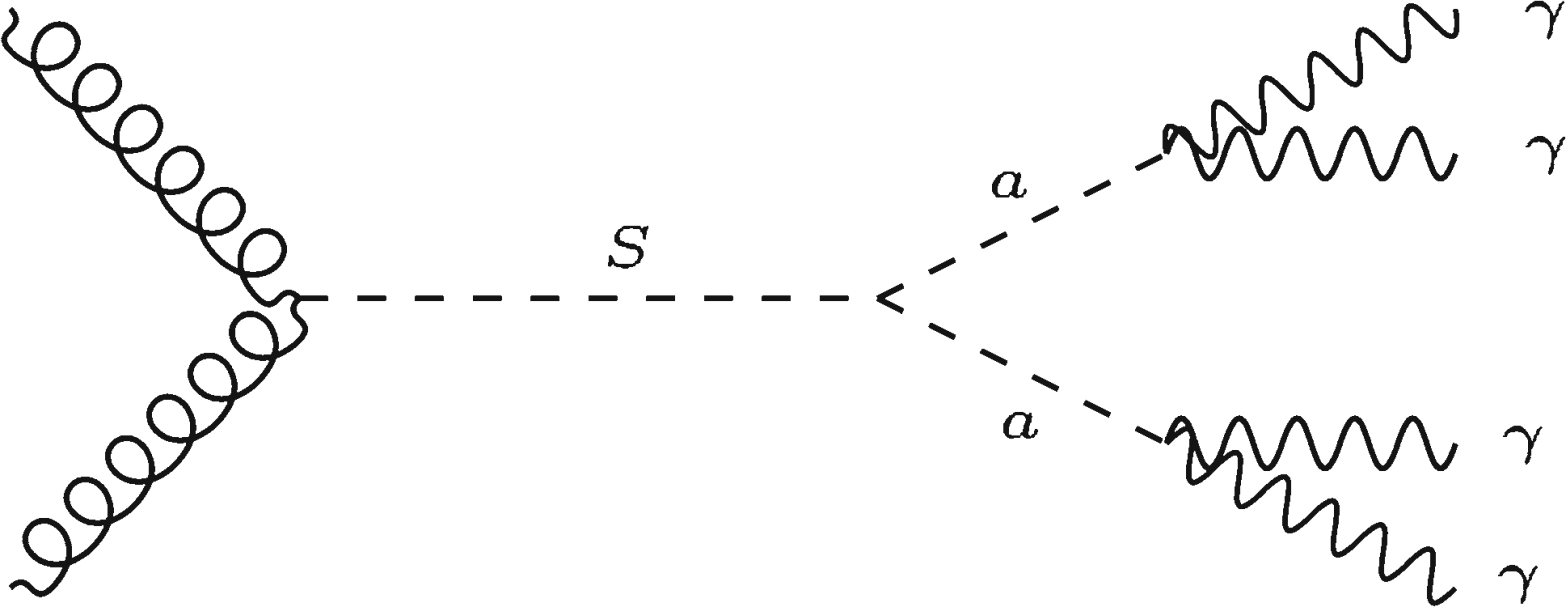
Feynman diagram illustrating the production of a scalar resonance *S* followed by the decay into collimated photon jets

Here, $m_S$ and $m_a$ are the masses of scalar and pseudoscalar, respectively, and $1/\Lambda$, 1 / *f*, $\lambda$ are coupling constants. An LHC process which is induced by these couplings is shown in Fig. 2. The five dimensionful parameters in Eq. (3) are a priori independent and can be extracted from the data. The position of the peak in the photon invariant mass peak determines $M_S$, and the signal cross section together with the decay width of *S* determines $\Lambda$ and $\lambda$. $m_a$ and *f* have to be chosen such that the photon jets pass as regular photons, which is non-trivial since the coupling *f* of a light pseudoscalar to photons is strongly constrained [13].

## Distinguishing photon jets from isolated photons

Consider a photon jet consisting of *N* collimated photons. A regular isolated photon corresponds to $N=1$ in this notation. A photon jet will be registered as a converted photon if at least one of the photons inside the jet converts and leaves a signal in the tracker. For a given conversion rate $p^\text {conv}$ for individual photons in a given jet, the probability that the photon jet appears as a converted photon is then given by

4 $$\begin{aligned} p_{N}^\text {conv}&= 1- \left[ 1 - p^\text {conv} \right] ^N. \end{aligned}$$

Obviously, the probability that the photon jet appears as an unconverted event is

5 $$\begin{aligned} p_{N}^\text {no-conv}&= 1-p_{N}^\text {conv}. \end{aligned}$$

For the moment, we neglect the possible issue arising from having more than two reconstructed tracks associated with the photon candidate, which could make the photon fail isolation criteria. We will come back to this point later.

Now consider a diphoton event^2^ with angular separation $\Delta R > 0.4$ so that they do not overlap. Microscopically, the event contains two photon jets, with the number of photons in them denoted by $N_1$ and $N_2$. From the experimental point of view, we distinguish three event categories, namely events with $i=0,1,2$ of the photon jets being reconstructed as converted photons. The probabilities $p^{(i)}_{(N_1 N_2)}$ for an event to fall into each of these categories depend on $N_1$ and $N_2$, and they thus offer a handle for distinguishing different theoretical models underlying a diphoton signal. It is easy to see that

6 $$\begin{aligned} p^{(0)}_{(N_1 N_2)}&= p_{N_1}^\text {no-conv} \, p_{N_2}^\text {no-conv},\end{aligned}$$

7 $$\begin{aligned} p^{(1)}_{(N_1 N_2)}&= p_{N_1}^\text {no-conv} \, p_{N_2}^\text {conv} + p_{N_1}^\text {conv} \, p_{N_2}^\text {no-conv}, \end{aligned}$$

8 $$\begin{aligned} p^{(2)}_{(N_1 N_2)}&= p_{N_1}^\text {conv} \, p_{N_2}^\text {conv}. \end{aligned}$$

In the following, we will in particular consider the prospects for distinguishing a real diphoton resonance, $(N_1 N_2) = (1 1)$, from models with $(N_1 N_2) = (1 2)$ or (22), which have been proposed in the literature as alternative explanations of the 750 GeV signal [11–16]. This discrimination is complicated by the fact that there is a significant number of SM background events in the signal region. Here we assume that all background events are of (11) type but we expect the results to remain similar for any other known background composition (see Appendix A for details).

Perhaps the simplest statistical way of approximately quantifying the model discrimination power is a Pearson $\chi ^2$ test based on the following $\chi ^2$ function:

9 $$\begin{aligned} \chi ^2(S,B)&= \frac{S^2}{B} \sum _{i=0}^2 \frac{\left( p^{(i)}_{(N_1^\text {true} N_2^\text {true})} - \, p^{(i)}_{(N_1^\text {test} N_2^\text {test})}\right) ^2}{ \frac{S}{B} \, p^{(i)}_{(N_1^\text {test} N_2^\text {test})} + p^{(i)}_{(11)}}. \end{aligned}$$

Here, *S* and *B* are the numbers of signal and background events, respectively, $(N_1^\text {true} N_2^\text {true})$ corresponds to the model we assume to be realized in nature, while $(N_1^\text {test} N_2^\text {test})$ describes the model we wish to test against. In other words, the question we are asking here is how likely it is that the hypothesis $(N_1^\text {test} N_2^\text {test})$ is accepted if the actual events are of type $(N_1^\text {true} N_2^\text {true})$. Obviously, the right hand side of Eq. (9) vanishes if $(N_1^\text {test} N_2^\text {test}) = (N_1^\text {true} N_2^\text {true})$.

**Fig. 3:**
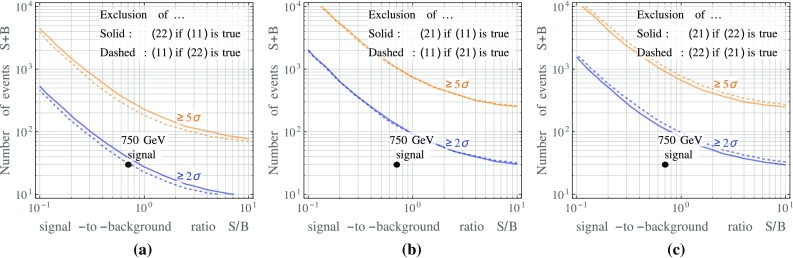
The expected number of diphoton events required to discriminate between different model hypotheses $(N_1 N_2)$ based on different conversion rates. Here, $N_1$ and $N_2$ are the multiplicities of the two photons or photon jets in the event. Results are shown as a function of the expected signal-to-background ratio. The sensitivities shown here are based on the likelihood ratio test discussed in Appendix B and include the $\eta$-dependence of the photon conversion probability

The two jets have different $p_T$ and pseudorapidities $\eta$, which lead to unequal $p^\text {conv}$ for the photons in different jets. To account for this, we take the $p_T$- and $\eta$-dependent conversion probabilities $p^\text {conv}(p_T, \eta )$ given in Ref. [17]. As the $p_T$-dependence of $p^\text {conv}(p_T, \eta )$ is weak for photons above 100 GeV, we neglect it in the following and work with $p^\text {conv}(\eta )$ depending only on the pseudorapidity. The value of $p^\text {conv}(\eta )$ in each $\eta$ bin is listed in Table 1 in Appendix B. The $\chi ^2$ function in Eq. (9) is generalized to also include a sum over the events in different bins (*jk*) labeled by the pseudorapidities $(\eta _j,\eta _k)$ of the two jets. The probabilities $p^{(i)}_{(N_1 N_2)}(\eta _j, \eta _k)$ for *i* conversions in an event in rapidity bin (*jk*) are given by Eqs. (6)–(8) using the appropriate $p^\text {conv}_{N_1}({\eta _1})$ and $p^\text {conv}_{N_2}({\eta _2})$ for each jet. Additionally, both terms in the numerator of Eq. (9) as well as the first term in the denominator must now be multiplied by $p_S^{jk}$, the probability in the respective true/test model for signal events to fall into that rapidity bin. Similarly the second term in the denominator must now be multiplied by the analogous probability $p_B^{jk}$ for background events. These probabilities $p_S^{jk}$ and $p_B^{jk}$ can be obtained by computing the differential cross sections for the signal and background $(N_1 N_2)$. We do so using MadGraph 5 v2.3.3 [28, 29], with a FeynRules / UFO [30] implementation of a simple $(N_1 N_2) = (11)$ model that augments the Standard Model with a scalar *S* and the effective couplings

10 $$\begin{aligned} \mathcal {L} \supset \frac{1}{\Lambda _g} \, S \, G_{\mu \nu } G^{\mu \nu } + \frac{1}{\Lambda _\gamma } \, S \, F_{\mu \nu } F^{\mu \nu }. \end{aligned}$$

Note that binning the data in pseudorapidity $\eta$ introduces some model dependence since the differential rapidity distribution will be different from model to model. We assume in the following that the $\eta$ distribution of the photon jets in models with $N_1, N_2 > 1$ is identical to the $\eta$ distribution of the isolated photons following from Eq. (10).

In our numerical results, we will go somewhat beyond the $\chi ^2$ test based on Eq. (9), and instead employ a slightly more sensitive likelihood ratio test, as discussed in Appendix B.

For a given *S* / *B*, we can now ask how many expected events $S+B$ in the signal region are needed to reject different hypotheses $(N_1 N_2)$ at the $2\sigma$ and $5\sigma$ level. The results are shown in Fig. 3. For *S* / *B* of order one, we see that at most a hundred events are necessary to distinguish the different hypotheses at the $2\sigma$ level. Discrimination between models of type (11) and (22) model requires fewer events than discrimination between the (21) and (11) or between the (21) and (22) scenarios. The reason is simply that the conversion probabilities Eqs. (6)–(8) for the two alternative hypotheses are more different in the former case. For the particular case of the excess observed around 750 GeV, the present data could already be sufficient to discriminated between the (11) and (22) hypotheses at the $2\sigma$ level, while more data would be needed to tell the (21) hypothesis apart from either (11) or (22) scenarios.

We see that photon conversion rates are a promising tool to distinguish between different new physics models in diphoton events once a signal is observed. However, also without an observed event excess, the different conversion probabilities for isolated photons and $N>1$ photon jets can be employed as an additional tool to discriminate photon jet signals from the background. In the following we illustrate this, again using the example of a search for a diphoton resonance in the mass range between 200 and 1500 GeV.

**Fig. 4:**
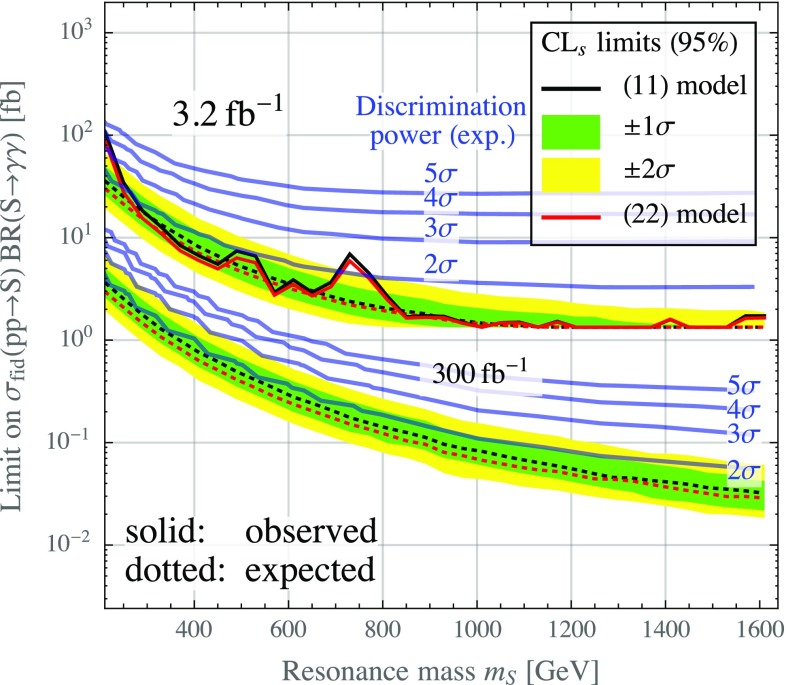
The expected (*dashed*) and observed (*solid*) 95 % CL$_s$ limits on a true diphoton signal (the (11) topology in our notation, *black curves* and Brazilian bands) and on a signal with two photon jets, each consisting of two photons (the (22) topology, *red curves*). To derive the observed limits, we have assumed that the $\eta$ distributions of the data and the conversion ratios follow the predictions from Monte Carlo simulations. We show results for an integrated luminosity of 3.2 fb$^{-1}$, corresponding to the data published in [1], and for an integrated luminosity of 300 fb$^{-1}$. The Brazilian bands were obtained using the CL$_s$ method [31, 32] as implemented in ROOT. The *blue contours* show the discrimination power between the (11) and (22) scenarios, defined here as the confidence level at which the (11) hypothesis can be rejected if the signal in the data consists of (22) photon jets

In Fig. 4, we show the expected and observed limits on such resonances in the ATLAS diphoton data with 3.2 fb$^{-1}$ of 13 TeV data [1], and the expected future limits in 300 fb$^{-1}$ of data. Note that the observed limits shown in Fig. 4 are based on the published ATLAS data, assuming that the $\eta$ distributions and the conversion rates (which are not public) follow the predictions from simulations. Comparing the limits on (11) resonances to those on (22) resonances, we observe a mild improvement in the latter case.

## Long-lived intermediate states

So far, we have assumed that the photon jets in a model with $N_1 > 1$ or $N_2 > 1$ form instantaneously at the primary interaction vertex. We consider now a more general scenario, where the intermediate particle *a* has a non-negligible proper lifetime $\tau$. In this scenario, *a* decays to photons only after traveling some macroscopic distance *x* in the inner detector. Since photon conversion cannot take place until the photons have been produced, the conversion probability for an individual photon is reduced. The reduction factor depends on many parameters, in particular on the distribution of material in the inner detector and on the efficiency for reconstructing tracks starting away from the beam axis. A full detector simulation is needed to determine this but a key ingredient is a knowledge of the radial dependence of the conversion probability $p^\mathrm{conv}$. In the following we outline two simplified approaches.

To obtain an intuitive understanding, it is useful to consider the highly simplistic assumption that the detector is homogeneous. The conversion probability then scales as $1 - x / L_t(\eta )$, where $L_t(\eta )$ is the total distance from the primary vertex to the edge of the tracker. The probability that at least one photon in an *N*-photon jet converts to an $e^+e^-$ pair inside the tracker is the probability that *a* decays between $x-\mathrm{d}x$ to *x*, and at least one of the *N* photons converts between *x* and $L_t(\eta )$, integrated over all *x* from 0 to $L_t(\eta )$. This is easy to compute and we find

11 $$\begin{aligned} p_N^\text {conv}(\eta , \tau )&= \int _0^{L_t(\eta )} \! \mathrm{d}x \, \frac{1}{\gamma \tau } \mathrm{e}^{-x / (\gamma \tau )} \nonumber \\&\quad \times \left[ 1 - \left( 1 - p^\text {conv}(\eta ) \left( 1 - \frac{x}{L_t(\eta )} \right) \right) ^N \right] , \end{aligned}$$

where $\gamma$ is the Lorentz boost of *a* and $p^\text {conv}(\eta )$ on the right hand side is, as in Sect. 3, the probability for a photon to convert between the point of production at the origin and the edge of the tracker at a distance $L_t(\eta )$. We have in particular, for $N=1,2$:

12 $$\begin{aligned}&p_1^\text {conv}(\eta , \tau ) = p^\text {conv}(\eta ) \left[ 1 - \left( 1 - \mathrm{e}^{-\frac{L_t(\eta )}{\gamma \tau }} \right) \frac{\gamma \tau }{L_t(\eta )} \right] , \end{aligned}$$

13 $$\begin{aligned}&p_2^\text {conv}(\eta , \tau ) = 2 p^\text {conv}(\eta ) \left[ 1 - \left( 1 - \mathrm{e}^{-\frac{L_t(\eta )}{\gamma \tau }} \right) \frac{\gamma \tau }{L_t(\eta )} \right] \nonumber \\&\quad - [p^\text {conv}(\eta )]^2 \left[ 1 - \frac{2 \gamma \tau }{L_t(\eta )} + 2 \left( 1 - \mathrm{e}^{-\frac{L_t(\eta )}{c\gamma \tau }} \right) \frac{\gamma ^2\tau ^2}{L_t^2(\eta )} \right] . \end{aligned}$$

Analogously, the probability for an *N*-photon jet to be detected without any of the photons converting is

14 $$\begin{aligned} p_N^\text {no-conv}(\eta , \tau )&= 1 - \mathrm{e}^{-L_c(\eta ) / (\gamma \tau )} - p_N^\text {conv}(\eta , \tau ). \end{aligned}$$

Here, the first two terms give the probability that the photon jet is detected at all, i.e. that *a* decays before reaching the calorimeter at a distance $L_c(\eta )$ from the primary vertex. Note that, because of this factor, $p_N^\text {conv}(\eta , \tau ) + p_N^\text {no-conv}(\eta , \tau ) < 1$.

For obtaining our numerical results, we model the conversion probability density as a function of the radial distance traveled using an approximate “two-zone” model based on Ref.  [33]. In the central region ($|\eta | <0.6$) there are 70 % conversions in the range $(0<r<15\,\mathrm{cm})$ and 30 % in $(15\,\mathrm{cm}< r < 40\,\mathrm{cm})$. In the forward region $(1.3<|\eta |<1.7)$ there are 65 % conversions in $(0<r<15\,\mathrm{cm})$ and 35 % in $(15\,\mathrm{cm}< r < 40\,\mathrm{cm})$, where *r* is the radial distance. Thus, $1 - x / L_t(\eta )$ is replaced by the above. The total conversion probability remains the same as before.

In an event with two photon jets, the boost factors $\gamma _1$, $\gamma _2$ for the two jets are in general different. Therefore, in the following numerical analysis, we fold the conversion probabilities with the distribution of $\gamma _1$, $\gamma _2$ in each $(\eta _1, \eta _2)$ bin, obtained from the same MadGraph simulation that determines the $(\eta _1, \eta _2)$ distribution (see Sect. 3). Afterwards, the analysis proceeds in the same way as in Sect. 3. In particular, the probability for zero, one or two of the photon jets in an event to convert are given by Eqs. (6)–(8), with the probabilities $p_N^\text {conv}$ and $p_N^\text {no-conv}$ on the right hand side replaced by the two-zone analog of Eqs. 11 and 14. The statistical analysis follows again the procedure described in Appendix B.

**Fig. 5:**
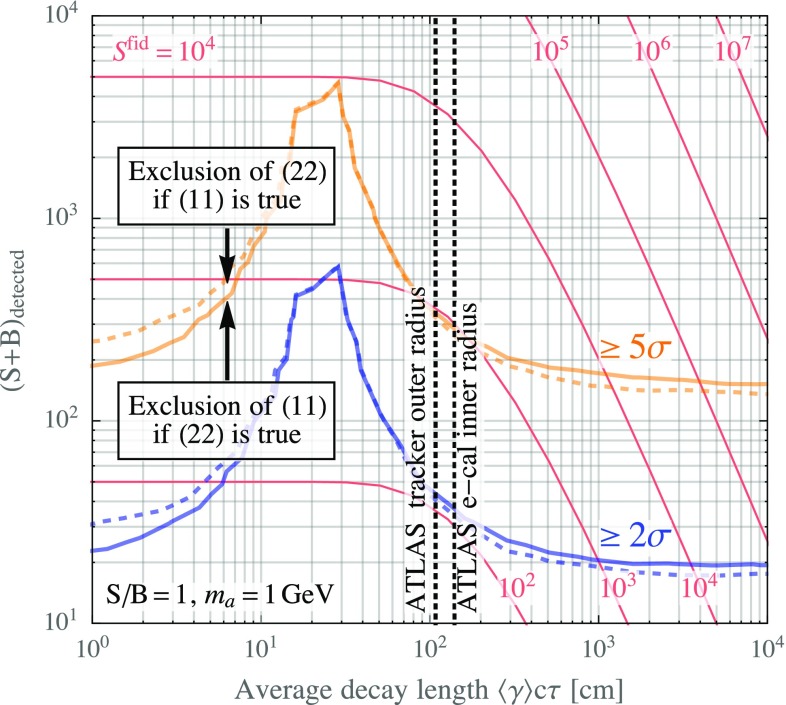
Number of events required to discriminate between a model predicting an $S \rightarrow \gamma \gamma$ signal and an alternative model predicting an $S \rightarrow aa \rightarrow 4\gamma$ final state. Results are shown as a function of the lifetime $\tau$ of the intermediate particle, multiplied by their average Lorentz boost $\left\langle \gamma \right\rangle$. For definiteness, we assume a signal-to-background ratio $S/B = 1$, and an *a* mass of 1 GeV. Note that $(S+B)_\mathrm{detected}$ only counts those events for which *a* decays before reaching the EM calorimeter. For $\langle \gamma \rangle c\tau$ larger than the inner e-cal radius, this is only a small fraction of the total number of required events, as indicated by the contours of constant total fiducial signal rate $S^\mathrm{fid}$

In Fig. 5, we show the number of expected signal events $S+B$ required to discriminate between models of (11) and (22) type as a function of the lifetime of the intermediate particle *a* in the (22) model. We take the mass of the heavy resonance decaying to photon jets to be 750 GeV, the mass of *a* to be 1 GeV, and we assume the model predicts a signal-to-background ratio $S/B = 1$. We emphasize that the vertical axis in Fig. 5 shows the expected number of *detected* diphoton events. Since for non-negligible $\tau$, only those events where both *a* particles decay before entering the calorimeter are detected, we also show for comparison the *total* number of signal events in the (22) case (red contours in Fig. 5).

As is to be expected, the discrimination power is best when $\left\langle \gamma \right\rangle \!\tau = 0$, and worsens for longer lifetimes because a decay away from the beam axis (but still well within the tracker) leads to a decreased conversion probability in the (22) model. When $\left\langle \gamma \right\rangle \!\tau \gtrsim L_c$, it is likely that *a* does not decay before reaching the calorimeter, so that events are no longer categorized as diphoton events. However, among those events which are detected, the fraction of converted events *increases* again. Since the vertical axis in Fig. 5 shows only the number $(S+B)_\text {detected}$ of *detected* diphoton events, the discrimination power based on $(S+B)_\text {detected}$ thus appears to *improve* again in this case. Note, however, that the condition $S/B=1$ requires a significantly larger cross section $\sigma _\text {fid} \, \text {BR}_{\gamma \gamma }$ when $\left\langle \gamma \right\rangle \!\tau$ is large.

**Fig. 6:**
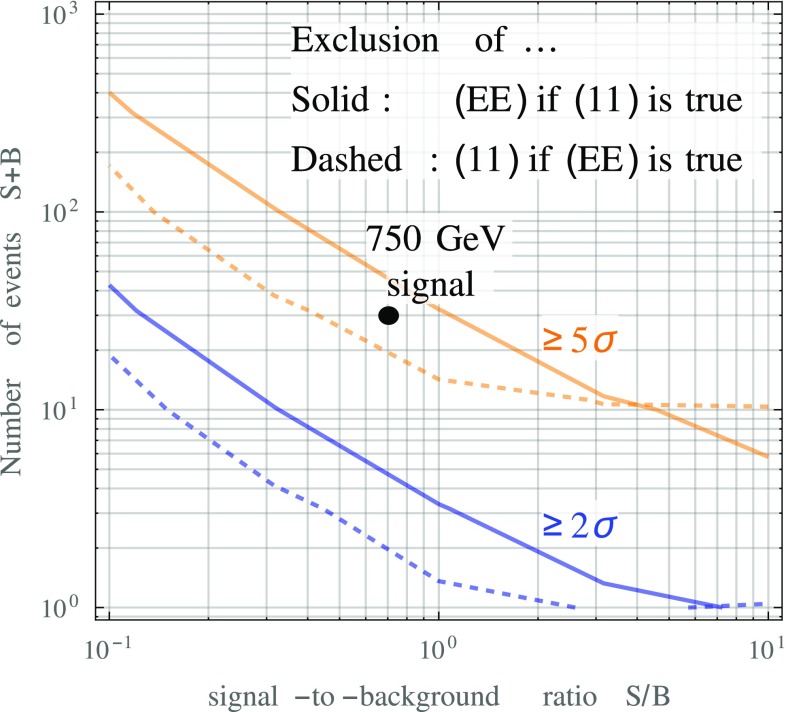
The expected number of diphoton events required to discriminate between a true diphoton signal ((11) model) and a signal of the form $p p \rightarrow S \rightarrow (A' \rightarrow e^+e^-) + (A' \rightarrow e^+e^-)$, where $A'$ is a dark photon. Results are shown as a function of the expected signal-to-background ratio. They are based on a likelihood ratio test, as discussed in Appendix B

## Dark photons

An interesting class of models that could in principle mimic a diphoton resonance signal are those where a new heavy particle *S* decays to two *dark photons* – the gauge bosons of a new $U(1)'$ gauge symmetry, often hypothesized in the context of dark matter models [22]. If the dark photon $A'$ is sufficiently light ($<\! m_\mu /2$), its dominant decay mode is $A' \rightarrow e^+ e^-$, so that the process $p p \rightarrow S \rightarrow (A' \rightarrow e^+e^-) + (A' \rightarrow e^+e^-)$ has the same final state as $p p \rightarrow S \rightarrow \gamma \gamma$, with both photons converting to $e^+ e^-$ pairs.^3^ While prompt $e^+e^-$ pairs will be vetoed in the photon reconstruction, if the dark photon lifetime is such that it mostly decays in the tracker, then these events could easily appear as a diphoton resonance.

At close inspections, the two topologies are of course different: first and foremost, $A'$ decays can only mimic converted photons. With good statistics, it should therefore be easy to tell an $A' A'$ signal apart from true isolated diphoton signal. This is indeed the case, as illustrated in Fig. 6: at a signal-to-background ratio of $\mathcal {O}(1)$, even a handful of events is enough to discriminate between the $A'$ model, denoted here as (EE), and a (11) type diphoton model. Even for a $5\sigma$ test, $\ll 100$ events are needed.

However, the difference in the apparent photon conversion probabilities is not the end of the story. For short-lived $A'$ decaying quasi-instantaneously, the $e^+e^-$ tracks in the (EE) model will come directly from the primary vertex, while the tracks from a converted $\gamma$ can originate at any radius *r* inside the tracker. Therefore, taking the radial distribution of the secondary vertices into account, the discrimination power can be boosted further. We have refrained from doing so in Fig. 6 to be conservative and because dark photons could also have a macroscopic decay length. In the latter case, the *r* distribution predicted by the (EE) model is much more similar to that of a converted $\gamma$ signal in the (11) scenario. Of course, small differences remain. For instance, an $A'$ decay can occur anywhere in the tracker, while photon conversion is only possible inside layers of detector material. Moreover, for $A'$ decay lengths comparable to the size of the tracker, the *r* distribution in the (EE) model is exponentially falling, while in the (11) model it is constant.

Note that, without inclusion of the *r* discrimination of the secondary vertices, the discrimination power depends on the laboratory frame $A'$ decay length $\gamma \tau$ only through the factor $[1 - \exp (L_c(\eta _1)/(\gamma \tau )] \, [1 - \exp (L_c(\eta _2)/(\gamma \tau )]$, which gives the probability that both $A'$ decays occur before the calorimeter (see Sect. 4). In other words, if *S* / *B* and $(S+B)_\text {detected}$ are fixed, as in Fig. 5, the discrimination power is independent of $\left\langle \gamma \right\rangle \!\tau$.

## Further observables

Photon reconstruction in the LHC detectors offers additional handles that could be used to further discriminate photon jets from isolated photons, and possibly pin down underlying structures like the multiplicity of photons inside the jet. In the following we briefly discuss the most promising ideas:

- The photon pairs coming from a boosted decay $a\rightarrow \gamma \gamma$ carry roughly equal energy. If only one of them converts, the ratio of track $p_T$ to calorimeter energy $E_\mathrm{cal}$ should differ substantially from one, the value expected for single photons. This is a very powerful variable that is not currently being used. An accurate measurement of the electron track $p_T$ is complicated by their relatively large $p_T\sim 100$ GeV and the fact that they only traverse part of the tracker, depending on where they convert. Therefore a sufficient number of events is needed such that the measurement can be made on those events where both electrons from the conversion are well reconstructed.
- When more than one photon inside a photon jets converts to $e^+e^-$, up to 2*N* tracks could be reconstructed for an *N*-photon jet. Such multiple conversions might be rejected by the standard photon reconstruction algorithms, for example in ATLAS [34] a cut is placed on the $p_T$ sum of tracks within $\Delta R=0.3$ of the photon candidate which are not associated with the photon candidate itself. Therefore we expect that the reconstruction efficiency for $N>1$ photon jets is reduced. However, once a resonance is found, events with larger track multiplicities can be explicitly searched for in loose photon samples to get additional information on the signal.
- Photon jet events will react differently from single photons to changes in the isolation criteria. While variables which mainly cut on nearby hadronic activity are insensitive to the photon multiplicity, those using electromagnetic calorimeter shower shapes could be very sensitive. In the ATLAS search for Higgs decays to pairs of photon jets [35], it was already shown that the variable $F_\mathrm{side}$, which considers the ratio of energies deposited in 3 vs. 7 bins centered on the highest bin, is very sensitive to the mass of the intermediate pseudoscalar $m_a$. A large change in efficiency of $F_\mathrm{side}$ in the signal region compared to the side bands would therefore be a strong indication of photon jets, and even give direct access to $m_a$. In this context, it is worth commenting on photon jets with $N>2$ constituent photons. At first glance it seems unlikely that such final states could successfully mimic an isolated photon signal, given how difficult it already is to sufficiently collimate two photons. However, one should also note that for $N>2$, the energy does not have to be distributed evenly between the photons. For instance, if one is significantly harder than the others, the energy deposit in the electromagnetic calorimeter would have a single peak structure such that rejection methods based on the shape of the calorimeter cluster would fail.

Each of these strategies can provide additional insight into the nature of a diphoton signal which might be discovered in the future, or more general into any new physics signals involving photons. Comparison with control regions and side bands can be used to verify that abnormal behavior of the photon candidates in the signal region is indeed due to photon jets and not just from e.g. QCD backgrounds.

## Conclusions

To summarize, we have discussed from a phenomenologist’s point of view how the conversion of photons to $e^+ e^-$ pairs inside the LHC detectors can be exploited to discriminate between final states involving isolated photons and events containing jets of multiple highly collimated photons. Such photon jets arise, for instance, when a light new particle is produced on-shell and decays to two photons. We have illustrated that, even with modest statistics, a resonance decaying to two isolated photons can be distinguished from a new particle decaying to two photon jets.

For instance, in the context of the possible 750 GeV resonance observed in ATLAS and CMS data, $\sim$30 events, are sufficient to make this distinction at the $2\sigma$ level, while $\mathcal {O}(100)$ events are required for a $5\sigma$ discrimination.

We have also illustrated how the sensitivity to photon jet signals mimicking a diphoton resonance depends mildly on the multiplicity of the photon jets. Finally, we have studied scenarios in which photon jets emerge at a macroscopic distance from the beam pipe in the decay of a long-lived intermediate particle.

We conclude that photon candidates in the LHC detectors offer an extremely rich substructure which can be exploited for highly efficient model discrimination. This substructure is theoretically well modeled and seems readily accessible experimentally. We hope that the results presented in this note will be useful in this endeavor.

## Figures and Tables

**Fig. 7:**
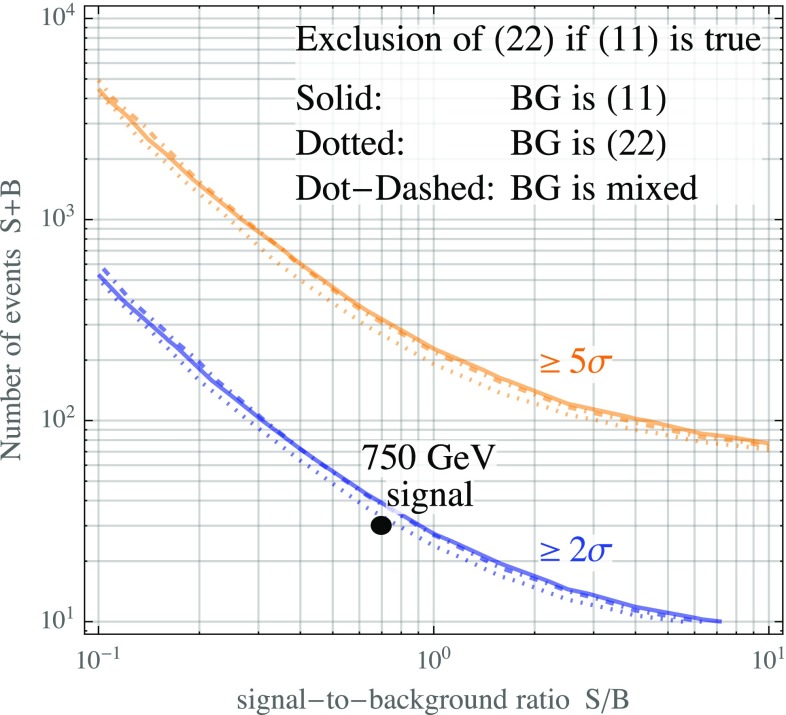
Discrimination power for different background compositions. For the mixed case we assume that half the background is of (11) type and the other half of (22) type

## Appendix A: Background dependence

The diphoton background usually has three components, which besides pairs of prompt photons includes events where either one or both photons are misidentified jets which essentially are due to neutral hadrons decaying to photon pairs. Thus the background too can have events of (12) and (22) type.

In the 750 GeV signal window, the jet contributions to the diphoton backgrounds are of order 10 % in CMS [2], and probably of the same order in ATLAS (see e.g. Fig. 7 in the supplemental material for Ref. [36]). Nevertheless we would like to stress that our method also works for different background composition, which could be relevant for applications in other search channels.

In Fig. 7 we see that the discrimination power is only marginally affected if the background composition is varied. Therefore as long as the background composition can be measured in control regions, the discrimination power will remain.

## Appendix B: Statistical procedure

Different theoretical models leading to a diphoton-like signature can be distinguished using a likelihood ratio test (see e.g. [37]). The test statistic is

B1 $$\begin{aligned} \text {LLR}(D, M_1, M_2) = \log \left( \frac{\mathcal {L}(D | M_1)}{\mathcal {L}(D | M_2)} \right) , \end{aligned}$$

where $\mathcal {L}(\text {data} | M_m)$ denotes the likelihood of the data *D* if the model hypothesis $M_m$ is true. A model hypothesis is characterized here by the multiplicities $(N_1 N_2)$ of the two photon jets in each signal event, and by the associated prediction $P_{M_m}^i$ for the event rate in the *i*th bin. The likelihood is given by

B2 $$\begin{aligned} \log \mathcal {L}(D | M_m) = \sum _i \left[ 2 ( D^i - P_{M_m}^i) + 2 D^i \log \frac{D^i}{P_{M_m}^i}\right] . \end{aligned}$$

In the simplest case, the bin index $i=0,1,2$ denotes the number of photon jets in the event that are reconstructed as converted photons. However, since the conversion probability depends on the rapidities $\eta _1$, $\eta _2$ of the photon jets and since the rapidity dependence is different for signal and background events, we bin the data also in $|\eta _1|$, $|\eta _2|$. This turns *i* into a multi-index (*ij* *k*), with $i = 0,1,2$ the number of converted photon jets, and *j*, *k* denoting the rapidity bins. We use 4 of the latter for each photon jet, as given in Table 1.

**Table 1:** Rapidity bins used in our analysis, together with the associated probabilities $p^\text {conv}$ for a single photon in a given bin to convert into an $e^+e^-$ pair

	bin boundaries in $\eta$	$p^\text {conv}(\eta )$
1	[0, 0.59]	0.25
2	[0.59, 1.35]	0.39
3	[1.52, 1.78]	0.54
4	[1.78, 2.4]	0.47

Note that this binning excludes the transition region between the barrel and the endcap. In the notation of Sect. 3, the number of predicted events $P_{M_m}^{(ijk)}$ for a model $M_m = (N_1 N_2)$ is given by

B3 $$\begin{aligned} P_{M_m}^{(ijk)} = B \, p_B^{jk} \, p_{(11)}^{(i)}(\eta _1^j, \eta _2^k) + S \, p_S^{jk} \, p_{(N_1 N_2)}^{(i)}(\eta _1^j, \eta _2^k). \end{aligned}$$

We wish to compute the expected confidence level at which model $M_2$ can be ruled out in favor of $M_1$, if $M_1$ is realized in nature. To do so, we generate $\mathcal {O}(10^4)$ sets $\{ \tilde{D}^{(ijk)} \}$ of pseudodata distributed according to model $M_2$ and compute the log-likelihood ratio $\text {LLR}(\tilde{D}, M_1, M_2)$ for each of them. We thus obtain the probability distribution function (PDF) of $\text {LLR}(\tilde{D}, M_1, M_2)$. We then compute also the log-likelihood ratio $\text {LLR}(M_1, M_1, M_2)$ for the case that the data $D^{(ijk)}$ equals the prediction $P_{M_1}^{ijk}$ of the assumed “true” model. Evaluating the cumulative distribution function (CDF) of $\text {LLR}(\tilde{D}, M_1, M_2)$ at the value $\text {LLR}(M_1, M_1, M_2)$ yields the desired confidence level for the exclusion of $M_2$.

While this Monte Carlo-based method for evaluating confidence intervals is very general and, by the Neyman–Pearson lemma, offers optimal discrimination power, it could be replaced by a much simpler $\chi ^2$ test. Namely, note that $-2 \log {\mathcal {L}(D | M_2)}$ follows a $\chi ^2$ distribution if the total number of events predicted by $M_2$ is not too small, The number of degrees of freedom of the $\chi ^2$ distribution is given by the number of bins. The expected confidence level at which model $M_2$ is disfavored if $M_1$ is true is thus given by the CDF of the $\chi ^2$ distribution, evaluated at $-2 \log {\mathcal {L}(M_1 | M_2)}$. If the number of events in each bin is $\gtrsim 10$, so that the Poissonian likelihood Eq. (B2) is well approximated by the Gaussian likelihood

B4 $$\begin{aligned} -2 \log \mathcal {L}^\text {Gauss}(D | M_m) = \sum _{i=0}^2 \sum _k \frac{(D^{(ijk)} - P_{M_m}^{(ijk)})^2}{P_{M_m}^{(ijk)}}, \end{aligned}$$

we recover the $\chi ^2$ from Eq. (9).

## Footnotes

1 To be more precise, the first layer of the EM calorimeter in ATLAS is very finely segmented with $\Delta \eta \approx 0.002-0.003$, and shower shape variables are used to suppress backgrounds from $\pi _0$ decays. So the actual bound on $m_a$ could be as low as 500 MeV, as argued in [11]. The main point here is that there is a region of parameter space where collimated photon jets can pass as single isolated photons, so the precise value of the limit is not important.

2 More precisely, an event with two reconstructed photon candidates which are well separated from each other.

3 We would like to thank Felix Yu for pointing out this possibility to us.
